# The Impact of Digital Inequities on Oropharyngeal Cancer Disparities in the United States

**DOI:** 10.1002/oto2.70113

**Published:** 2025-04-09

**Authors:** David J. Fei‐Zhang, Achilles A. Kanaris, Camaren M. Cuenca, Sydney A. Fleishman, Jill N. D'Souza, Anthony M. Sheyn, Daniel C. Chelius, Jeffrey C. Rastatter

**Affiliations:** ^1^ Northwestern University Feinberg School of Medicine Chicago Illinois USA; ^2^ Baylor College of Medicine Houston Texas USA; ^3^ Department of Otolaryngology Louisiana State University Health Sciences Center, Division of Pediatric Otolaryngology, Children's Hospital of New Orleans New Orleans Louisiana USA; ^4^ Department of Pediatric Otolaryngology Le Bonheur Children's Hospital Memphis Tennessee USA; ^5^ Department of Otolaryngology–Head and Neck Surgery University of Tennessee Health Science Center Memphis Tennessee USA; ^6^ Department of Pediatric Otolaryngology St. Jude Children's Research Hospital Memphis Tennessee USA; ^7^ Department of Otolaryngology–Head and Neck Surgery Pediatric Thyroid Tumor Program and Pediatric Head and Neck Tumor Program, Baylor College of Medicine, Texas Children's Hospital Houston Texas USA; ^8^ Department of Otolaryngology–Head and Neck Surgery Northwestern University Feinberg School of Medicine Chicago Illinois USA; ^9^ Division of Pediatric Otolaryngology, Ann & Robert H. Lurie Children's Hospital of Chicago Chicago Illinois USA

**Keywords:** digital divide, digital inequity, head and neck, oral cavity cancer, oral squamous cell carcinoma, oropharyngeal cancer, oropharyngeal squamous cell carcinoma, social determinants of health

## Abstract

**Objective:**

To assess associations of digital inequity with oropharyngeal cancer (OPC) prognostic and care outcomes in the United States while adjusting for traditional social determinants/drivers of health (SDoH).

**Study Design:**

Retrospective cohort study.

**Setting:**

United States.

**Methods:**

In total, 70,604 patients from 2008 to 2017 were assessed for regression trends in long‐term follow‐up period, survival, prognosis, and treatment across increasing overall digital inequity, as measured by the Digital Inequity Index (DII). DII is based on 17 census‐tract level variables derived from the American Community Survey and Federal Communications Commission. Variables were categorized as infrastructure‐access (ie, digital‐related variables) or sociodemographic (ie, education, income, and disability status) and weighted‐averaged into a composite score.

**Results:**

With increasing DII, decreases in length of follow‐up (10.22%, 32.9‐29.5 months; *P* < .001) and survival (8.93%, 19‐17.3 months; *P* < .001) were observed. Affordability of internet access displayed the largest influence, followed by device access and internet‐service availability. Compared to OPC patients with low digital inequity, high digital inequity was associated with increased odds of diagnosing more than one malignant tumor (odds ratio [OR] 1.01, 95% CI 1.01‐1.03; *P* = .012) and advanced staging (OR 1.01, 95% CI 1.00‐1.02; *P* = .034), while having decreased odds of receiving indicated chemotherapy (OR 0.98, 95% CI 0.97‐0.99; *P* < .001), radiation therapy (OR 0.98, 95% CI 0.97‐0.99; *P* < .001), or primary surgery (OR 0.98, 95% CI 0.97‐0.99; *P* < .001).

**Conclusion:**

Digital inequities contribute to detrimental trends in OPC patient care and prognosis in the United States. These findings can inform strategic discourse targeted against the most pertinent disparities in the modern‐day environment.

As the incidence of oropharyngeal cancers (OPC) projects to continually rise over the coming years,^1^, ^2^, ^3^, ^4^, ^5^ such trends have been driven by a multitude of factors. These include multiple clinical and behavioral aspects related to human papillomavirus (HPV) infection,^6^ tobacco usage,^4^ and sexual behaviors.^7^ Additionally, numerous social factors related to social determinants/drivers of health (SDoH), including those related to socioeconomic status, race‐ethnicity status, education, and others, have been large proponents in conferring OPC disparities.^8^, ^9^, ^10^

Intertwined with these clinical and social factors of OPC outcomes, digital access has been imperative toward informing, affecting, and modifying these characteristics for OPC providers and patients. Whether that would be through providing educational materials on promoting prophylactic behaviors, such as HPV vaccination or healthy sexual practices,^11^, ^12^, ^13^ facilitating health care appointments,^14^, ^15^ or other aspects of streamlining communication between physician and patient, varied access to digital resources has proven to be a great divider in determining who receives proper OPC care and diagnosis. However, in contrast to the numerous investigations into traditional SDoH factors and digital inequities across other head‐neck cancers,^16^, ^17^, ^18^, ^19^ there is scant investigation into how digital factors associate with differences in OPC prognosis and care.

In light of these gaps, large‐data tools, such as the Digital Inequity Index (DII), provide a means for assessing multiple aspects of internet access and electronic device infrastructure on a national scale, alongside adjusted‐for traditional, nondigital SDoH factors that would indirectly affect digital inequities. Through utilizing the DII measures, this study aims to assess OPC care and prognostic outcomes across the United States. We hypothesize that, after accounting for traditional SDoH associations, digital resource differences would have significant independent associations with poorer follow‐up and survival while impacting aspects of care associated with decreased indicated treatment receipt.

## Methods

This retrospective cohort study follows the Strengthening the Reporting of Observational Studies in Epidemiology (STROBE) reporting guideline. Prior institutional review board, ethics committee approval, or waiver of informed consent were not needed as per Northwestern University's institutional policy as the databases queried for this study consisted of publicly available, deidentified data.

### Data Set Sources

**Figure 1:**
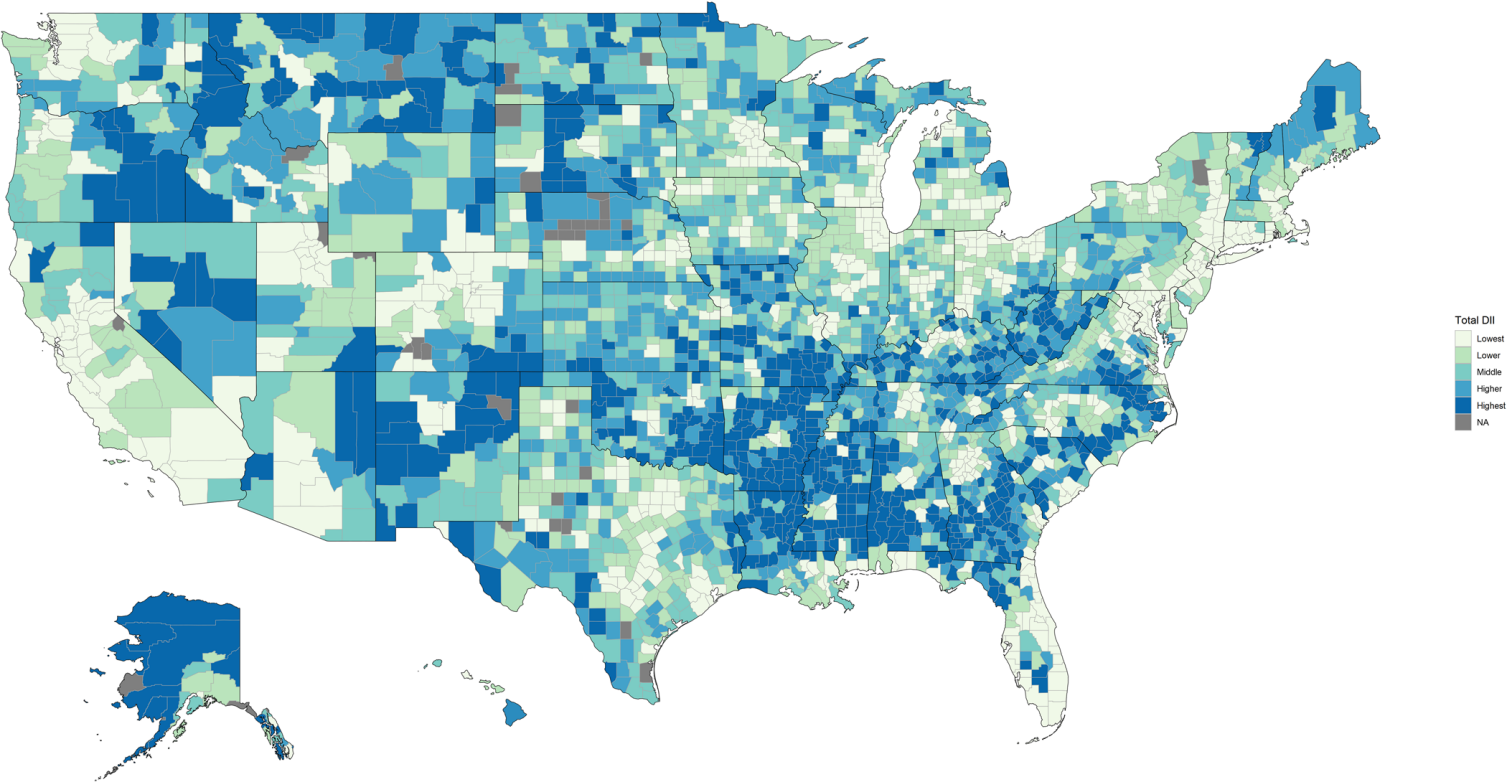
Distribution of total Digital Inequity Index scores across the United States.

Based on 17 census‐tract level variables derived from the 2018 American Community Survey (ACS) 5‐year estimates from 2008 to 2017 and Federal Communications 14th Broadband Report, the DII was formulated utilizing extracted variables from these sources and grouped into two DII subcategories of “infrastructure‐access”—comprised of the measures representing “households without a desktop or laptop,” “without access to nonmobile broadband,” “without access to broadband: DSL,” “without access to broadband: cable,” “without access to broadband: fiber,” “without access to broadband: fiber,” “without access to broadband: terrestrial fixed wireless,” “without a mobile or nonmobile internet subscription of any type,” “without an internet subscription of cable, fiber, or DSL,” “without a broadband subscription in households making $20,000 or less,” “without a broadband subscription in households making $20,000 to $74,999,” and “without a broadband subscription in households making $75,000 or more”—and “sociodemographic”—comprised of “25+ aged people without high school diploma,” “25+ aged people without an associate's degree or higher,” “25+ aged people without a bachelor's degree or higher,” “below poverty level within the last 12 months,” “below 150% of poverty level within the last 12 months,” and “disability status pertaining to cognitive, ambulatory, or self‐care difficulties.” Ranked scores were then assigned to each variable and adjusted by tract population to calculate weighted‐mean scores on the county level within their respective DII subcategories. A total composite score was formulated based on the combined means of the two subcategories to account for nondigital, sociodemographic confounders. DII scores were then arranged into ordered classes by natural break (Jenks) classification by comparing the sum of squared deviations between classes to each array mean and utilize a goodness of variance fit. These five classes were then labeled as “lowest,” “lower,” “middle,” “higher,” and “highest” (Figure 1). These methods were adapted from the Rural Indiana Stats group and the creation of the Indiana state‐localized Digital Divide Index.^20^

The National Cancer Institute‐Surveillance‐Epidemiology‐End Results Program (NCI‐SEER) database contains publicly available data sets of patient variables, pathological characteristics, treatment modalities, and prognostic outcomes. Months under surveillance represents a length‐of‐care measurement reflecting the active follow‐up a patient receives for their primary malignancy up until the last provider interaction. Months survival represents active follow‐up until patient suffers a mortal outcome. Staging is based on SEER‐designated variables labeled as “stage IV,” “distant [expansion],” or “distal [expansion]” and recoded under American Joint Committee on Cancer, 6th Edition (AJCC‐6) classifications. The number of primary tumors is defined by SEER multiple primaries with identical histological type and dictates its anatomical localization as “in situ,” “localized,” or “within the local cancerization field” to delineate it from other aspects pertaining to nodal/metastasis. Primary surgery occurrence represents whether patients received surgery for their primary malignancy. Similarly, chemotherapy and radiation therapy receipt.

DII scores were abstracted and matched to SEER‐patient data based on county‐of‐residence at the time of diagnosis.

### Population Definitions

The SEER database was queried for adult (20+ years) patients diagnosed with OPC between 2008 and 2017 using the International Classification of Diseases for Oncology, Third Edition (ICD‐O‐3) topographic codes (C10.0‐10.9).

### Statistical Analysis

Demographics tables were grouped by total DII scores delineated by natural break (Jenks) classifications of “lowest,” “lower,” “middle,” “higher,” and “highest.”

Follow‐up time/surveillance period and survival period within each primary malignancy were analyzed by total DII score and DII subcategory scores. DII scores were split into relative, equivalently sampled quintiles based on actual DII scores within each primary malignancy. The relative‐DII quintiles were delineated by “<20,” “20 to 39.99,” “40 to 59.99,” “60 to 79.99,” and “80 to 99.99,” representing their relative percentiles per malignancy type (eg, within disease A, patients with the lowest DII scores are grouped into the “<20” quintile group). Among these total and DII‐theme quintiles, differences between the mean months under surveillance and survival period for the lowest and highest DII‐scored quintiles were calculated. Trend significance was assessed by linear regression across relative‐DII quintiles for both continuous measures, and box plots were generated to assess the median, interquartile range (IQR), and 1.5 times the IQR. Mean values were also calculated per relative quintile group. Survival months within respective primary malignancies were analyzed similarly as months under surveillance. However, after separating patients into relative‐DII quintiles within each respective primary malignancy, patients who were alive/lost upon last follow‐up were excluded to extract patients who were dead upon last follow‐up.

Logistic regression was used to assess primary surgery occurrence, advanced staging at time of diagnosis, and the receipt of radiation therapy across DII quintiles. A two‐sided *P*‐value < .05 was set as the threshold of statistical significance.

## Results

A total of 70,604 patient cases of primary OPC were extracted from SEER. Patients in our study were more likely to be 45 to 64 years of age (51.4%, n = 36,333), male (73.3%, n = 51,790), white (77.3%, n = 54,617), and from the west (49.7%, n = 35,117). Patients were stratified by total DII score into the categories of “lowest” (71.2%, n = 50,293), “lower” (14.2%, n = 10,033), “middle” (7.7%, n = 5435), “higher” (3.7%, n = 2626), and “highest” (3.1%, n = 2217). DII scores distributed across the United States are displayed in Figure 1. Notably, although patients from the south were 25.7% of our study population, they comprised 91.7% (n = 2032) of patients in the “highest” total DII group and 79.8% (2095) of patients in the “higher” total DII group. Additional patient demographic and clinical characteristics are displayed in Table 1.

**Table 1:** Clinicodemographics by Digital Inequity Index (DII) Score

		Total DII category					
Characteristic	N	Lowest total DII, N = 50,293 (71%)	Lower total DII, N = 10,033 (14%)	Middle total DII, N = 5435 (7.7%)	Higher total DII, N = 2626 (3.7%)	Highest total DII, N = 2217 (3.1%)	*P*‐value
Age	70,604						<.001
20‐44 y		2570 (5.1%)	478 (4.8%)	249 (4.6%)	122 (4.6%)	83 (3.7%)	
45‐64 y		25,661 (51%)	5186 (52%)	2870 (53%)	1417 (54%)	1199 (54%)	
65‐84 y		19,248 (38%)	3873 (39%)	2063 (38%)	982 (37%)	844 (38%)	
85+ y		2814 (5.6%)	496 (4.9%)	253 (4.7%)	105 (4.0%)	91 (4.1%)	
Sex	70,604						.073
Male		36,804 (73%)	7346 (73%)	4008 (74%)	1981 (75%)	1651 (74%)	
Female		13,489 (27%)	2687 (27%)	1427 (26%)	645 (25%)	566 (26%)	
Race	70,604						<.001
White		38,348 (76%)	7885 (79%)	4473 (82%)	2152 (82%)	1759 (79%)	
Black		3279 (6.5%)	1300 (13%)	621 (11%)	298 (11%)	378 (17%)	
Hispanic		4251 (8.5%)	492 (4.9%)	198 (3.6%)	58 (2.2%)	45 (2.0%)	
Asian or Pacific Islander		3669 (7.3%)	219 (2.2%)	71 (1.3%)	102 (3.9%)	5 (0.2%)	
Unknown		515 (1.0%)	85 (0.8%)	32 (0.6%)	13 (0.5%)	10 (0.5%)	
Native American		231 (0.5%)	52 (0.5%)	40 (0.7%)	3 (0.1%)	20 (0.9%)	
Region	70,604						<.001
Midwest		3132 (6.2%)	2607 (26%)	912 (17%)	249 (9.5%)	16 (0.7%)	
Northeast		8070 (16%)	2008 (20%)	355 (6.5%)	0 (0%)	0 (0%)	
South		8137 (16%)	2744 (27%)	3130 (58%)	2095 (80%)	2032 (92%)	
West		30,954 (62%)	2674 (27%)	1038 (19%)	282 (11%)	169 (7.6%)	
TNM combined staging	67,807						.985
Stages I‐III		26,804 (56%)	5317 (55%)	2927 (55%)	1390 (55%)	1178 (55%)	
Stage IV and above		21,460 (44%)	4312 (45%)	2349 (45%)	1116 (45%)	954 (45%)	
No. of primary tumors by Dx	64,544						.020
1		34,642 (75%)	6804 (75%)	3775 (76%)	1796 (76%)	1561 (77%)	
2 or more		11,467 (25%)	2313 (25%)	1169 (24%)	562 (24%)	455 (23%)	
Primary surgery performed	69,868						.002
No surgery		24,376 (49%)	5008 (50%)	2708 (51%)	1311 (51%)	1080 (50%)	
Surgery		25,498 (51%)	4909 (50%)	2642 (49%)	1255 (49%)	1081 (50%)	
Radiation therapy performed	70,604						.010
No therapy		19,975 (40%)	3985 (40%)	2172 (40%)	1041 (40%)	966 (44%)	
Therapy		30,318 (60%)	6048 (60%)	3263 (60%)	1585 (60%)	1251 (56%)	
Chemotherapy performed	70,604						.024
No therapy		26,817 (53%)	5312 (53%)	2952 (54%)	1384 (53%)	1250 (56%)	
Therapy		23,476 (47%)	4721 (47%)	2483 (46%)	1242 (47%)	967 (44%)	
Vital status on last follow‐up	70,604						<.001
Alive		33,086 (66%)	6076 (61%)	3256 (60%)	1515 (58%)	1236 (56%)	
Dead		17,207 (34%)	3957 (39%)	2179 (40%)	1111 (42%)	981 (44%)	
3‐y mortality	46,756						<.001
Alive, >3 y		18,288 (56%)	3436 (50%)	1834 (49%)	850 (47%)	705 (45%)	
Dead, ≤3 y		14,552 (44%)	3390 (50%)	1894 (51%)	951 (53%)	856 (55%)	
5‐y mortality	36,612						<.001
Alive, >5 y		8978 (35%)	1708 (31%)	864 (29%)	389 (27%)	336 (26%)	
Dead, ≤5 y		16,424 (65%)	3788 (69%)	2107 (71%)	1072 (73%)	946 (74%)	

Abbreviation: Dx, diagnosis.

### Trends in Months Under Surveillance and Survival

**Figure 2:**
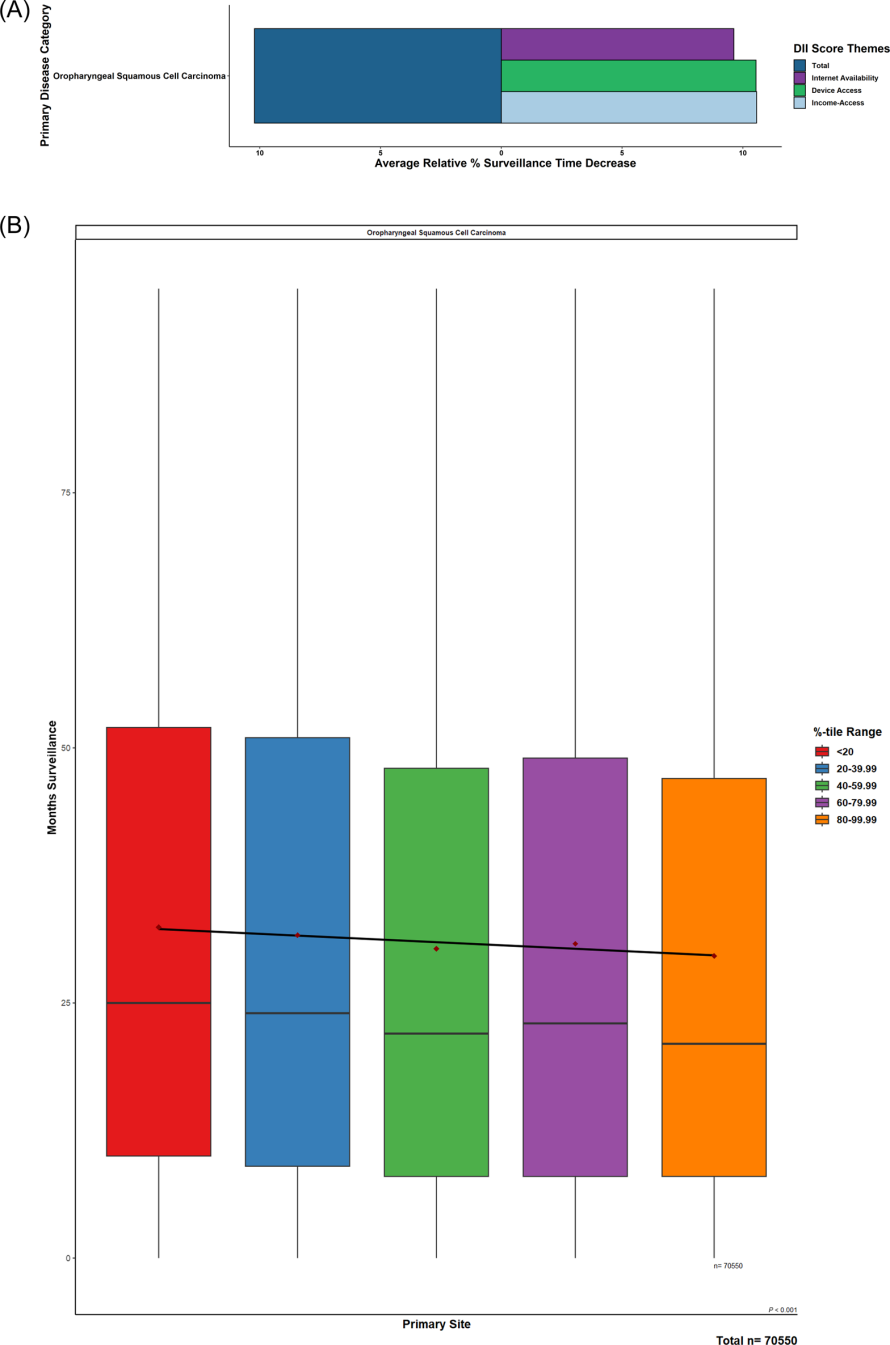
Relative trends of months surveillance with increasing Digital Inequity Index (DII) scores. (A) Oropharyngeal cancer patients of increasing total DII levels were regressed across surveillance periods. (B) Barplots highlighted relative magnitude decreases of DII components across these trends.

As total DII scores increased (ie, digital inequity increased), a significant decrease (*P* < .001) in mean months of surveillance was observed when comparing patients in the lowest total DII quintile (32.9 months) to those in the highest total DII quintile (29.5 months), which was equivalent to an average decrease of 10.22%. Affordability of internet access was the largest contributor, followed closely by device access and then internet‐service availability (Figure 2).

**Figure 3:**
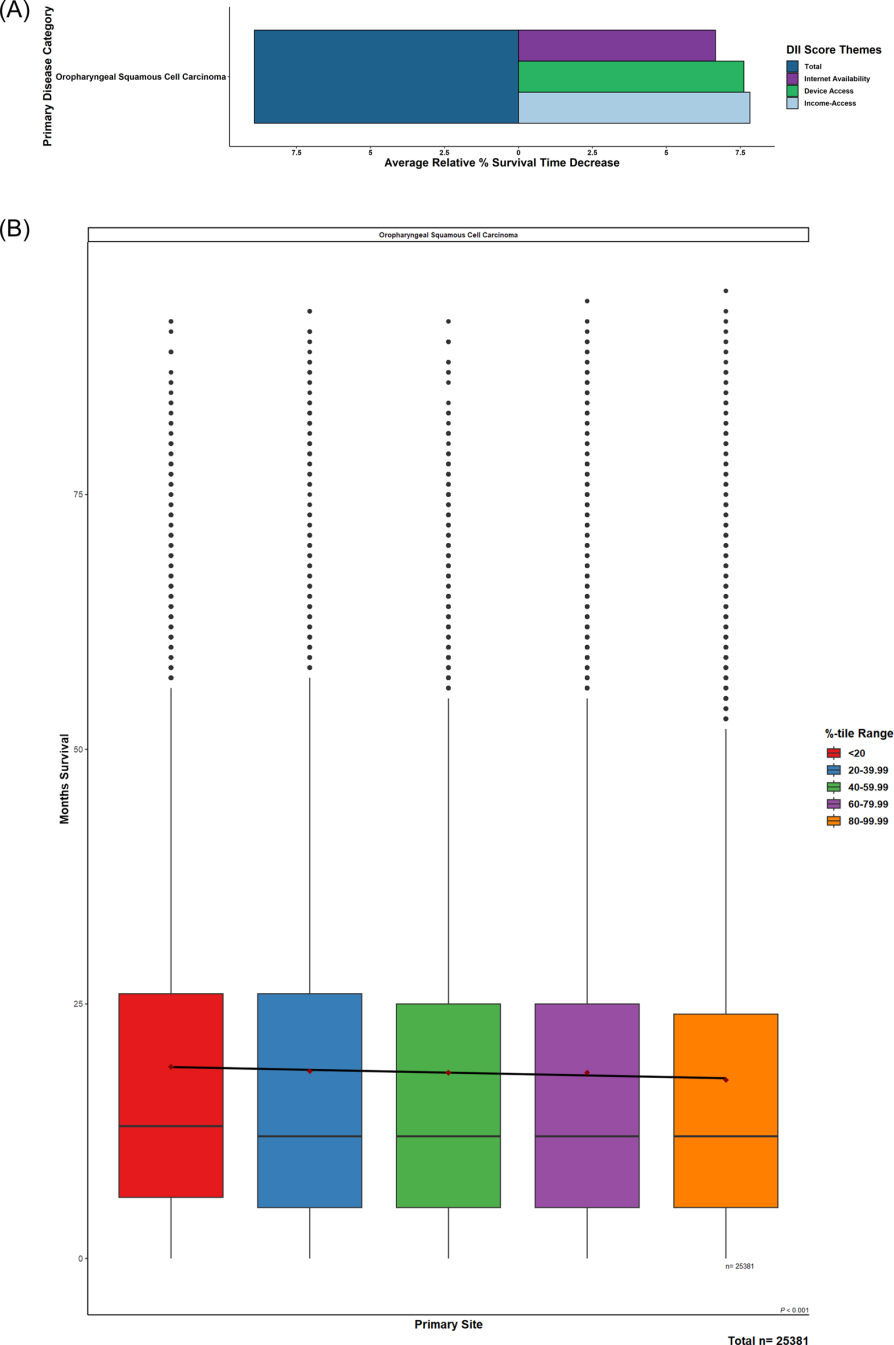
Relative trends of months survival with increasing Digital Inequity Index (DII) scores. (A) Oropharyngeal cancer patients of increasing total DII levels were regressed across survival periods. (B) Barplots highlighted relative magnitude decreases of DII components across these trends.

Similarly, for survival period, mean months of survival exhibited decreasing trends as digital inequity/DII scores increased (*P* < .001) when comparing patients in the lowest total DII quintile (19 months) to those in the highest total DII quintile (17.3 months), equal to an average decrease of 8.93%. Again, affordability of internet access was observed to be the largest contributor, with device access and internet‐service availability following (Figure 3).

### Trends in Preliminary Diagnosis and Treatment

With regard to disease staging, compared to patients in the lowest DII score quintile (ie, the least digital inequity), patients in the highest DII score quintile (ie, the highest digital inequity) were associated with increased odds of being diagnosed with more than one primary tumor (odds ratio [OR] 1.01, 95% CI 1.01‐1.03; *P* = .012) and with advanced staging at preliminary diagnosis (OR 1.01, 95% CI 1.00‐1.02; *P* = .034) (Table 2).

**Table 2:** Advanced Staging on Preliminary Presentation With Increasing Digital Inequity Index (DII) Scores

Outcome	Characteristic	OR	95% CI	*P*‐value
Advanced staging on preliminary presentation	Total DII	1.01	1.00, 1.02	.034
More‐than‐one primary tumor on preliminary presentation	Total DII	1.01	1.01, 1.03	.012

Abbreviation: OR, odds ratio.

Regarding received treatment, OPC patients in the highest DII score quintile were associated with decreased odds of receiving indicated chemotherapy (OR 0.98, 95% CI 0.97‐0.99; *P* < .001), radiation therapy (OR 0.98, 95% CI 0.97‐0.99; *P* < .001), and surgical resection (OR 0.98, 95% CI 0.97‐0.99; *P* < .001) (Table 3).

**Table 3:** Multimodal Indicated Treatment Receipt With Increasing Digital Inequity Index (DII) Scores

Outcomes	Characteristic	OR	95% CI	*P*‐value
Primary surgery	Total DII	0.98	0.97, 0.99	.001
Radiation therapy	Total DII	0.98	0.97, 0.99	<.001
Chemotherapy	Total DII	0.98	0.97, 0.99	<.001

Abbreviation: OR, odds ratio.

## Discussion

Our study serves as the first foray into digital inequity as it relates to OPC outcomes through national, large‐data approaches. By utilizing the DII, we showcased that there are independent associations between poor digital access and worse outcomes in OPC patients while controlling for sociodemographic factors. The results of our investigation show that with increasing digital inequity as assessed by the DII, OPC patients were found to have significant decreases in surveillance and survival time. Furthermore, OPC patients were at increased odds of being diagnosed with more than one malignant tumor and were less likely to receive indicated treatment, whether chemotherapy, radiation therapy, or surgical resection.

Although OPC rates are expected to continue to rise in an older population, the HPV vaccine provides an opportunity to decrease its prevalence in younger cohorts. Given the absence of effective screening measures for OPC, education on preventative measures, such as HPV vaccination, is paramount.^21^ The HPV vaccine has been shown to decrease the prevalence of oral HPV infections, the precursors to HPV‐related OPC, prompting the US Food and Drug Administration to expand the indications for the HPV‐9 valent vaccine for the prevention of OPC in 2020.^4^, ^7^, ^22^ Based on recent HPV vaccination rates and data from the SEER database, Zhang et al project that OPC incidence rates will decrease in patients aged 36 to 55 years between 2018 and 2045, with an estimated 6334 OPC cases being prevented as a result of vaccination.^23^

Our research illuminates an opportunity to increase this impact. As previously discussed, internet access is directly related to the delivery of HPV information and subsequent vaccination.^14^, ^24^, ^25^, ^26^ With regard to our study, patients from the south were disproportionately represented in high DII quintiles, and henceforth affected in our outcomes and treatment receipt trends. This aligns with prior research demonstrating that the south accounts for the lowest HPV vaccination rates with correlated increased rates of cervical cancer when compared to other regions in the United States.^27^, ^28^, ^29^, ^30^

Composed of many rural communities, this trend of digital inequity in the south is one that had been observed and deemed a critical issue by the federal government. The US Federal Communications Commission, under the Telecommunications Act of 1996, has a goal of universal service for all Americans with Congress, approving $42 billion in 2023 to accelerate the expansion of access to rural communities through initiatives like the ReConnect program.^31^ However, reports from 2023 indicate that the program has yet to set specific goals for what it should achieve or how it will measure its progress.^32^ Research such as ours can inform where and how these programs should target their efforts to dually address the digital divide and resultant health inequities that can be addressed with health information dissemination and telehealth services.

As SDoH research continues to illuminate the social inequities that contribute to health disparities in cancer treatment and outcomes, digital inequity must be included as a relevant factor in a rapidly evolving digital landscape. The DII addresses limitations found in existing social determinant tools while ensuring robustness through rigorous data sources such as the ACS/US Census and the Federal Communications Commission Broadband Report.^8^, ^33^, ^34^, ^35^, ^36^, ^37^ By employing similar quantitative methodologies seen in established indices, the DII provides verified, detailed demographic information across its 17 digital and sociodemographic variables. This approach facilitates comprehensive assessments of social disparities at the county or census level and allows for weighted scoring of both total and subcategory variables. Additionally, the multivariate framework of the DII supports quantitative analyses, including investigations into disparities related to cancers like OPC, bolstering its applicability in diverse research contexts.

The foremost strengths of this study lie in its use of the DII as a novel and comprehensive index of determinants of digital inequities while controlling for sociodemographic disparities. Additionally, utilizing these data with that from the SEER database allowed for a large, modern‐day sample with level‐of‐care and prognostic outcome information.

This study is not without its limitations. Primarily, the DII and SEER data only overlap from 2008 to 2017, which necessitates future census and patient data that provide more current representations of health outcomes and social disparities in the digital inequity context. Additionally, the specific SEER data set utilized does not include HPV status, restricting the ability to complete subgroup analyses. The standalone SEER database lacks a comprehensive set of clinical variables needed to fully characterize our findings, necessitating the use of additional paid, SEER‐Medicare‐linked databases to obtain more detailed information on operative details and treatment modalities. Furthermore, the retrospective nature of this study limits it to conclusions only on associations, not causation, between digital inequity and OPC.

## Conclusion

Through utilizing a large‐data index tool of the DII, this study showcased how national differences in digital access and electronic device availability are independently associated with disparities in OPC prognosis and care. These results build upon prior studies of other pathologies by highlighting the pervasive effects of amalgamated and specific digital access factors while accounting for traditional SDoH drivers. From this higher‐level study, this showcases the DII as a foundation for future inquiry to inform both clinical practice and public policy surrounding the importance of addressing modern‐day determinants of OPC disparities.

## Author Contributions

**David J. Fei‐Zhang**, conceptualization, data acquisition, data analysis, data interpretation, validation, figure/table generation, drafting, critical revisions, supervision; **Achilles A. Kanaris**, conceptualization, data interpretation, validation, figure/table generation, drafting, critical revisions; **Camaren M. Cuenca**, data interpretation, drafting, critical revisions; **Sydney A. Fleishman**, data interpretation, drafting, critical revisions; **Jill N. D'Souza**, conceptualization, data interpretation, validation, critical revisions, supervision; **Anthony M. Sheyn**, conceptualization, data interpretation, validation, critical revisions, supervision; **Daniel C. Chelius**, conceptualization, data interpretation, validation, critical revisions, supervision; **Jeffrey C. Rastatter**, conceptualization, data interpretation, validation, critical revisions, supervision.

## Disclosures

### Competing interests

The authors declare that there is no conflict of interest.

### Funding source

The authors received no financial support for the research, authorship, and/or publication of this article.
